# The Lung Microbiome in Moderate and Severe Chronic Obstructive Pulmonary Disease

**DOI:** 10.1371/journal.pone.0047305

**Published:** 2012-10-11

**Authors:** Alexa A. Pragman, Hyeun Bum Kim, Cavan S. Reilly, Christine Wendt, Richard E. Isaacson

**Affiliations:** 1 Department of Medicine, University of Minnesota, Minneapolis, Minnesota, United States of America; 2 Department of Veterinary and Biomedical Sciences, University of Minnesota, St. Paul, Minnesota, United States of America; 3 Division of Biostatistics, School of Public Health, University of Minnesota, Minneapolis, Minnesota, United States of America; 4 Department of Medicine, VA Medical Center, Minneapolis, Minnesota, United States of America, and for the FORTE Study Group; Leiden University Medical Center, The Netherlands

## Abstract

Chronic obstructive pulmonary disease (COPD) is an inflammatory disorder characterized by incompletely reversible airflow obstruction. Bacterial infection of the lower respiratory tract contributes to approximately 50% of COPD exacerbations. Even during periods of stable lung function, the lung harbors a community of bacteria, termed the microbiome. The role of the lung microbiome in the pathogenesis of COPD remains unknown. The COPD lung microbiome, like the healthy lung microbiome, appears to reflect microaspiration of oral microflora. Here we describe the COPD lung microbiome of 22 patients with Moderate or Severe COPD compared to 10 healthy control patients. The composition of the lung microbiomes was determined using 454 pyrosequencing of 16S rDNA found in bronchoalveolar lavage fluid. Sequences were analyzed using mothur, Ribosomal Database Project, Fast UniFrac, and Metastats. Our results showed a significant increase in microbial diversity with the development of COPD. The main phyla in all samples were Actinobacteria, Firmicutes, and Proteobacteria. Principal coordinate analyses demonstrated separation of control and COPD samples, but samples did not cluster based on disease severity. However, samples did cluster based on the use of inhaled corticosteroids and inhaled bronchodilators. Metastats analyses demonstrated an increased abundance of several oral bacteria in COPD samples.

## Introduction

Chronic obstructive pulmonary disease (COPD), a chronic inflammatory lung disorder characterized by non-reversible airflow limitation, is presently the third-leading cause of death in the United States. Cigarette smoking is the principal cause of COPD in industrialized nations, but only approximately 20% of adults with substantial tobacco exposure develop clinically significant COPD. Some patients with advanced COPD are prone to exacerbations, which are characterized by worsening dyspnea, wheezing, cough, and sputum production [1]. Therefore, COPD remains a heterogeneous disease with respect to disease susceptibility and progression. The pathogenesis of COPD likely involves many as-yet undescribed mediators of inflammation, with bacterial infection or colonization likely playing a role [2].

Traditional microbial culture techniques have demonstrated that approximately 50% of COPD exacerbations are associated with pathogens such as *Streptococcus pneumoniae*, *Haemophilus influenzae,* and *Moraxella catarrhalis*. These organisms can often be found colonizing the airways of COPD patients between exacerbations [3]. The term lung microbiome has been used to describe this community of organisms inhabiting the lung. Since many of these bacteria persist in the airways of patients with COPD, their presence may promote a chronic inflammatory state that drives COPD pathogenesis.

In the past, studies of the microbiome relied on culture-based systems. New techniques for describing the microbiome using 16S rRNA pyrosequencing have allowed us to taxonomically classify and describe the human microbiome without the biases inherent in microbial culture techniques [4]–[6]. Charlson *et al.* demonstrated the presence of 16S rDNA sequences in the bronchoalveolar lavage fluid (BALF) of healthy volunteers. The authors noted a significant correlation between each subject’s oropharyngeal and lung microbiomes, but no consistent lung-specific microbiome was found across subjects. The authors concluded that the normal lung microbiome consisted of organisms that gained access to the lower respiratory tract through microaspiration or bronchoscopic carryover [7].

Accurate descriptions of the microbiome have allowed us to study the interactions between the microbiome and the host immune system. The microbiome in early childhood may play a role in the development of asthma [8], while specific components of the microbiome are associated with chronic asthma in adulthood [9]. As has been described by the “hygiene hypothesis” for asthma pathogenesis, exposure to a normal commensal microbiome may promote immune tolerance, which is necessary for normal immune system maturation and control of inflammation [10]. An analogous process may be at play in the pathogenesis of other lung diseases that involve the interplay between the microbiome, immune tolerance, and infection. For instance, Herbst *et al.* have shown that the normal mouse microbiome is necessary for normal maturation, recruitment and control of allergic airway inflammation [11], while Ichinohe *et al.* showed that the mouse microbiome helps regulate the immune function necessary to respond to Influenza A virus infection [12].

The lung microbiomes of healthy smokers as well as patients with COPD have been described and reviewed [2], [13]. Huang *et al.* showed that patients experiencing COPD exacerbations requiring ventilator support and broad-spectrum antibiotics maintained diverse lung microbiomes [14]. Erb-Downward *et al.* showed that the microbiomes of 2 patients with Moderate or Severe COPD had lower bacterial diversity scores than healthy smokers and healthy non-smokers. They described a core COPD lung microbiome that included *Pseudomonas, Streptococcus, Prevotella, Fusobacterium, Haemophilus, Veillonella,* and *Porphyromonas*. In addition, the lung microbiomes of Very Severe COPD patients were sampled intensively at the time of transplantation. They noted striking differences in the microbiomes at adjacent lung sites, driven by the dominance of *Pseudomonas, Haemophilus*, and *Stenotrophomonas* in each sample [15]. Sze *et al*. evaluated the lung tissue microbiomes of 8 Very Severe COPD patients at the time of lung transplantation. They noted increased bacterial diversity in the COPD patients compared to controls. COPD patients had an increase in the phylum Firmicutes, attributable to an increase in *Lactobacillus* [16].

We hypothesize that alterations in the COPD lung microbiome and/or its interactions with the host immune system may lead to disordered immune tolerance and the development of an inflammatory state that accelerates the progression of COPD. We undertook our study to evaluate the lung microbiomes of a large number of patients with Moderate or Severe COPD and compare them with the microbiomes of control patients. We chose to use COPD patients without a recent exacerbation to determine if the microbiome became more diverse during disease progression and limit the microbiome-altering effects of steroids and antibiotics.

## Results

Thirty-two samples from 3 groups (10 Control samples, 14 Moderate COPD samples, and 8 Severe COPD samples) were submitted for 454 pyrosequencing. Over 460,000 sequences were obtained, with each sample averaging 14,451 sequences after trimming and quality control filtering (Table 1). The number of operational taxonomic units (OTUs) observed at 97% identity ranged from 3-119. There were statistically significant differences in the numbers of sequences obtained from the Control and COPD groups as indicated by a *p*-value of 0.0326. Using the Bonferroni method for *post hoc* comparisons we found that this is driven by the smaller number of sequences in the Severe COPD group, as compared to the Moderate COPD group. There was no difference in the number of OTUs obtained per sample between the groups (*p* = 0.36). Shannon and Simpson (1-D) diversity indices demonstrated that the Severe COPD group was the most diverse, followed by the Moderate COPD group; the Control group was the least diverse. These differences in the diversity indices were significantly different among the Control and COPD groups (Shannon *p* = 0.0082, Simpson *p* = 0.0167 by the Kruskal Wallace test) and in *post hoc* comparisons using the Bonferroni method we found that the differences were driven by differences among the Control and Severe COPD groups (for the Simpson index the difference between the control group and the Severe COPD group just missed the cutoff). However, when we statistically control for the effect of age we find that there is not a significant difference among the groups (the Shannon index *p-*values for Control and Moderate COPD patients and Control and Severe COPD patients are *p* = 0.49 and *p* = 0.73, respectively, while the *p-*values for the Simpson index are *p* = 0.30 and *p* = 0.89, respectively), but age is associated with diversity (*p* = 0.0163 for the Shannon index and *p* = 0.0062 for the Simpson index). Moderate COPD patients were older than severe COPD patients (*p* = 0.0241 by Wilcoxon’s test).

**Table 1:** Patient Characteristics and Sequencing Results.

Sample	Age	Gender	Smoking Status*	Trimmed Sequences	OTUs	Shannon Index	Simpson (1-D) Index
Control 4	29	Male	Nonsmoker	5,845	34	0.11	0.03
Control 5	48	Female	Nonsmoker	19,756	17	0.72	0.50
Control 7	50	Female	Nonsmoker	11,102	16	0.02	0.00
Control 8	42	Male	Smoker	21,438	50	0.34	0.11
Control 9	28	Male	Nonsmoker	20,010	47	0.39	0.13
Control 10	46	Female	Smoker	19,824	73	1.35	0.68
Control 12	46	Male	Smoker	22,012	20	0.34	0.17
Control 14	33	Female	Nonsmoker	9,340	45	0.62	0.33
Control 19	48	Male	Nonsmoker	14,210	29	1.20	0.55
Control 23	25	Male	Smoker	23,422	14	0.30	0.15
Control Average	39.5±9.7			16,696±6105#	34.5±18.2	0.54±0.44†	0.27±0.24†
Moderate 18	71	Male	Nonsmoker	15,670	14	0.78	0.49
Moderate 22	69	Male	Nonsmoker	12,280	45	0.83	0.45
Moderate 43	63	Male	Nonsmoker	12,996	27	0.15	0.05
Moderate 55	76	Male	Nonsmoker	25,174	99	1.77	0.70
Moderate 60	73	Female	Nonsmoker	13,470	109	2.51	0.83
Moderate 74	66	Male	Nonsmoker	10,898	28	1.63	0.72
Moderate 86	78	Male	Nonsmoker	20,166	41	1.76	0.76
Moderate 93	76	Male	Nonsmoker	5,070	26	0.74	0.41
Moderate 95	77	Male	Nonsmoker	11,221	93	2.74	0.87
Moderate 112	64	Male	Nonsmoker	14,276	44	1.48	0.57
Moderate 138	73	Male	Nonsmoker	13,828	49	1.86	0.78
Moderate 146	75	Male	Nonsmoker	19,391	119	2.47	0.87
Moderate 184	55	Male	Nonsmoker	16,900	11	0.02	0.00
Moderate 190	60	Male	Nonsmoker	17,468	47	1.62	0.68
Moderate COPD Average	69.7±7.1§			14,915±4843	53.7±34.7	1.45±0.85	0.58±0.28
Severe 13	62	Male	Nonsmoker	11,325	35	1.38	0.66
Severe 52	70	Female	Nonsmoker	8,383	56	2.59	0.90
Severe 64	65	Male	Nonsmoker	11,130	45	2.00	0.79
Severe 72	65	Male	Nonsmoker	12,175	42	1.09	0.45
Severe 73	57	Male	Nonsmoker	9,654	89	2.01	0.76
Severe 85	64	Male	Nonsmoker	10,201	89	2.73	0.87
Severe 153	59	Male	Nonsmoker	11,570	85	2.09	0.78
Severe 166	59	Male	Nonsmoker	12,214	3	0.00	0.00
Severe COPD Average	62.6±4.2§			10,832±1330	55.5±28.7	1.74±0.89	0.65±0.30
All COPD Average	67.1±7.0			13,430±4376#	54.4±33.4	1.55±0.85†	0.61±0.28†

* All COPD patients were non-smokers for at least 6 months prior to study entry.

# Fewer sequences were obtained from COPD samples than from Control samples (*p* = 0.0326); this association was driven by the lower number of sequences in the Severe COPD (compared to Moderate COPD) group. This was not associated with a difference in the number of OTUs obtained.

† COPD samples were significantly more diverse than Control Samples (*p* = 0.0082 Shannon, *p* = 0.0167 Simpson). This severity effect disappears when we control for age.

§ Moderate COPD patients are older than Severe COPD patients (*p* = 0.0241).

Rarefaction curves were calculated for all samples, and showed that with very few exceptions, additional sampling would not have provided additional OTUs (Figure S1). A Venn diagram was created to illustrate the similarities between each group (Figure S2). All sequences in each subject group were combined, with 285 OTUs observed in the Control group, 412 OTUs in the Moderate COPD group, and 253 OTUs in the Severe COPD group. Significant overlap was observed between the Severe and Moderate COPD groups, with 56% and 34% of OTUs shared between the two, respectively. In contrast, only 17% of OTUs were shared between Control and Moderate COPD, and only 23% of OTUs were shared between Control and Severe COPD groups. Only 6.3% of all OTUs were found in common in all 3 groups.

**Figure 1:**
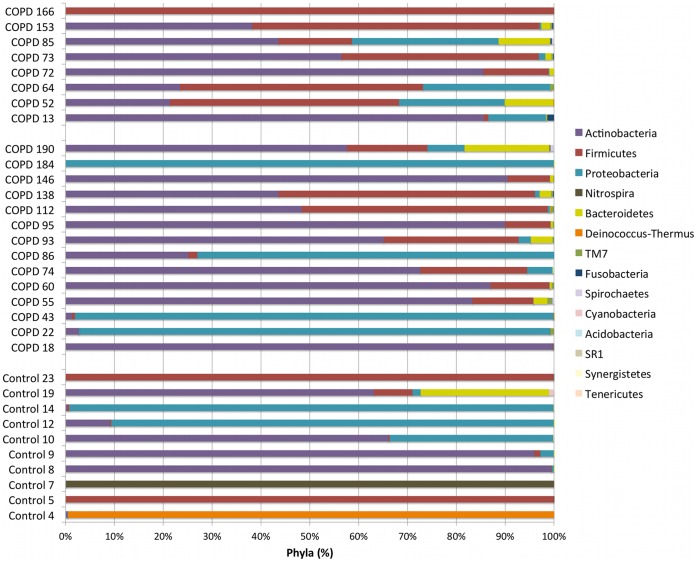
Taxonomic Identification at the Phylum Level. All sequences were submitted to RDP Classifier for taxonomic identification with a bootstrap cutoff of 50%. Taxonomic results at the phylum level are displayed for each sample with Control samples at the bottom, Moderate COPD samples in the middle, and Severe COPD samples at the top. The legend is organized from most (top) to least abundant (bottom) phyla.

Sequences were submitted to RDP Classifier for taxonomic identification with a bootstrap cutoff of 50%. Phylum-level classification for each sample is provided in Figure 1. The most common phylum in all samples was Actinobacteria, followed by Firmicutes, Proteobacteria, Nitrospira, and Bacteroidetes. Most control samples contained a mix of Actinobacteria, Firmicutes and Proteobacteria. Two samples (Control 4 and 7), both with low diversity indices and a moderate number of OTUs, were unexpectedly dominated by the phyla Deinococcus-Thermus or Nitrospira, respectively. The corresponding genera *Deinococcus* and *Nitrospira* have been isolated from numerous environmental sites, but have not been isolated from humans. The Moderate COPD group contained mostly Actinobacteria and Proteobacteria. Two samples (Moderate 43 and 184) with low diversity indices were dominated by Proteobacteria. The Severe COPD group contained mostly Actinobacteria and Firmicutes. One sample (Severe 166) with a low diversity index was dominated by Firmicutes. It appeared that Severe COPD samples contained more Firmicutes and less Actinobacteria and Proteobacteria than the Moderate COPD samples; however, statistical analysis did not demonstrate a significant association.

**Figure 2:**
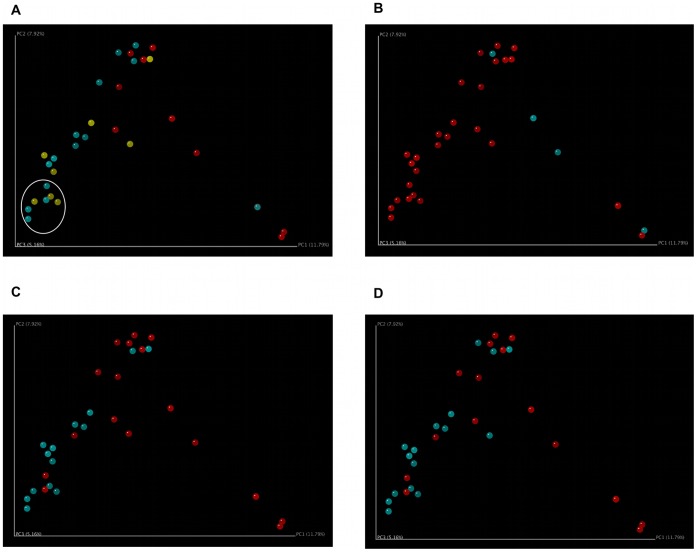
Principal Coordinate Analysis Demonstrates Clustering of COPD Samples, Inhaled Corticosteroid Users, and Inhaled Bronchodilator Users. Principal coordinate analysis was performed using mothur and Fast UniFrac, and the results for principal coordinates 1 and 2 are shown. A. Control, Moderate COPD, and Severe COPD. Control samples (red) cluster separately from Moderate COPD (blue) and Severe COPD (yellow) samples. Moderate and Severe COPD samples do not cluster separately. Seven COPD samples that separate the most from the control samples are circled and designated “left lower quadrant” (LLQ) samples for further analysis. B. Smokers and Non-Smokers. Smokers (blue) do not cluster separately from Non-Smokers (red). All of the COPD patients had been non-smokers for at least 6 months prior to bronchoscopy. C. Inhaled Corticosteroid Users and Non-Users. Inhaled corticosteroid users (blue, 14 of 22 COPD patients) are more likely to cluster near the intersection of principal coordinates 1 and 2 than non-users (red). D. Inhaled Bronchodilator Users and Non-Users. Inhaled bronchodilator users (blue, 16 of 22 COPD patients) are more likely to cluster near the intersection of principal coordinates 1 and 2 than non-users (red). All patients who received inhaled corticosteroids also received inhaled bronchodilators.

In order to evaluate the similarities between our samples, principal coordinate analysis (PCoA) was performed using Fast UniFrac. This analysis revealed clustering of control and COPD samples. No separation between Moderate COPD and Severe COPD samples was observed (Figure 2A). We identified 7 COPD samples that clustered most distinctly from the control samples and labeled them “left lower quadrant” samples (LLQ, circled). These 7 samples were almost evenly divided between Moderate and Severe samples and included COPD 55, 73, 85, 93, 138, 146, and 153.

Using the clinical information available on these subjects, we also analyzed the data for clustering based on other clinical parameters. We were specifically interested in the potential effects of tobacco exposure or immunosuppressant drugs such as steroids on the lung microbiome. All of the COPD subjects were non-smokers for at least 6 months prior to bronchoscopy, but 4 of our control subjects were smokers. PCoA did not demonstrate any clustering among the 4 control subjects who were active tobacco users at the time of bronchoscopy (Figure 2B). We then turned our attention to the potential effect of steroid use on the microbiome. None of the COPD patients had used systemic steroids in the 2 months prior to bronchoscopy. However, 14 of the 22 COPD patients were using inhaled corticosteroids (ICS) while none of the control subjects were using ICS or systemic steroids. PCoA demonstrated clustering of the ICS users (Figure 2C). We were also interested in the effects of other lung medications, including inhaled bronchodilators (IBD). Of the 16 COPD patients using IBD, 14 were also using ICS. PCoA did demonstrate clustering of IBD users (Figure 2D), but given the high degree of overlap between the ICS- and IBD-using populations, we are unable to determine which medication drives this association. We also analyzed the PCoA data for clustering based on age, gender, percent of lung tissue with emphysema (determined based on CT scanning), and theophylline use. No clustering based on these clinical parameters was observed (Figure S3A–D).

To determine the taxa responsible for the clustering observed on PCoA analysis, we used Metastats to detect differentially abundant features between samples. We compared COPD vs. Control samples, ICS users vs. non-users, IBD users vs. non-users, and samples in the left lower quadrant (LLQ, circled in Figure 2A) vs. all others (Table 2). In each comparison, separate analyses were performed at each taxonomic level. In order to control the false discovery rate at 10%, we reported only taxa with q-values <0.10 (it transpires that all of these comparisons also had *p*-values less than 0.05). We primarily focused our discussion on organisms that were differentially abundant in 3 or more of our 4 analyses, and on organisms that were differentially abundant at multiple corresponding taxonomic levels.

**Table 2:** Metastats Analysis of Differential Abundance.

Phylum	Class	Order	Family	Genus
Actinobacteria	Actinobacteria	↑Coribacteriales^AD^	↑Coriobacteriaceae^A^	↑Atopobium^A^
				↑Cryptobacterium^ABC^
				↑Olsenella^A^
		↓Rubrobacterales^C^	↓Rubrobacterineaceae^C^	↓Rubrobacter^C^
		Actinomycetales	Nocardioidaceae	↑**Nocardioides^ABC^**
			↑Micrococcaceae^AC^	↓Arthrobacter^A^
				↑Rothia^AC^
			Propionibacteriaceae	↓Propionibacterium^B^
			↑Kineosporiaceae^D^	
			↑Cellulomonadaceae^C^	↑Tropheryma^C^
			↑Actinomycetaceae^A^	↑Actinomyces^A^
			↓Geodermatophilaceae^AC^	↓Modestobacter^AC^
			↓Nakamurellaceae^ABC^	↓Humicoccus^ABC^
		Bifidobacteriales	Bifidobacteriaceae	↑Bifidobacterium^A^
Firmicutes	Bacilli	Bacillales	Bacillalaceae	↑**Pontibacillus** ^BC^
			↓Thermoactinomycetaceae^ABC^	↓Thermoactinomyces^ABC^
		↑Lactobacillales^AD^	↑Streptococcaceae^AD^	↑Streptococcus^A^
			↑Aerococcaceae^ABC^	↑Abiotrophia^ABC^
	Erysipelotrichi	↑Erysipelotrichales^A^	↑Erysipelotrichaceae^A^	↑Bulleidia/↑Solobacterium^A^
	Negativicutes	Selenomonadales	↑Veillonellaceae^A^	↑Dialister^A^
				↑Veillonella^A^
				↑Selenomonas^A^
				↑Centipeda^A^
	Clostridia	Clostridiales	↑Lachnospiraceae^A^	↑Catonella^A^
				↑Oribacterium^A^
				↑Butyrivibrio^A^
			↑Eubacteriaceae^A^	↑Eubacterium^A^
			Clostridiaceae	↑Anaerosporobacter^A^
				↑**Clostridium** ^BC^
				↓Anaerobacter^A^
			Peptostreptococcaceae	↓Sporacetigenium^BC^
				↑Peptostreptococcus^A^
			Unclassified Clostridales	↑Howardella^D^
			Clostridales incerta sedis	↑Parvimonas^A^
↑Fusobacteria^A^	↑Fusobacteria^A^	↑Fusobacteriales^A^	↑Leptotrichiaceae^A^	↑Leptotrichia^A^
			↑Fusobacteriaceae^A^	↑Fusobacterium^A^
Proteobacteria	α-proteobacteria	↑Rhodospirillales^A^	↑Bradyrhizobiaceae^BC^	↑**Balneimonas** ^ABC^
		Sphingomonadales	↑Sphingomonadaceae^B^	
	β-proteobacteria	↑Neisserales^A^	Neisseriaceae	↑Kingella^ABC^
	γ-proteobacteria	↑**Aeromonadales** ^ABC^	↑**Aeromonadaceae** ^ABC^	↑**Aeromonas** ^AB^
		Pseudomonadales	Pseudomonadaceae	↑Azomonas^BC^
		Enterobacteriales	Enterobacteriaceae	↑Serratia^A^
				↓Citrobacter^ABC^
				↑Enterobacter^BC^
		↑Cardiobacteriales^D^		
	δ-proteobacteria	↑**Desulfobacterales** ^ABC^	↑**Desulfobulbaceae** ^ABC^	↑Desulfobulbus^AC^
		↑Desulfovibrionales^D^		
	↑ε-proteobacteria^A^	↑Campylobacteriales^A^	↑Campylobacteriaceae^A^	↑Campylobacter^A^
Acidobacteria	↑**Group 3** ^ABC^			
Bacteroidetes	Bacteroidia	Bacteroidales	Porphyromonadaceae	↑Dysgonomonas^ABC^
				↑Tannerella^A^
			Prevotellaceae	↑Prevotella^A^
				↑Hallella^A^
			Unclassified Bacteroidales	↑Phocaeicola^BCD^
			↑Bacteroidales incerta sedis^BCD^	
	Flavobacteria	Flavobacteriales	Flavobacteriaceae	↑Planobacterium^BC^
				↑Capnocytophaga^AC^
Synergistetes	↑Synergistia^D^	↑Synergistales^D^		
↑SR1^BC^				↑**SR1 genera incertae sedis** ^BC^
↑TM7^A^				↑TM7 genera incerta sedis^A^

A Change in abundance in COPD (compared to Control).

B Change in abundance in 14 subjects using inhaled corticosteroids (compared to all other COPD samples and controls).

C Change in abundance in 16 subjects using inhaled bronchodilators (compared to all other COPD samples and controls).

D Change in abundance in 7 COPD samples in the left lower quadrant (compared to all other COPD samples and controls).

**Bolded Taxa** were differentially expressed in Severe COPD (compared to Moderate COPD).

At the phylum level, several changes were noted. The anaerobic gram-negative phylum Fusobacteria was increased in the COPD samples, and this increase was reflected at all taxonomic levels down to the genera *Leptotrichia* and *Fusobacterium*, two bacteria found in the oral flora [17]. The candidate phyla SR1 and TM7 and their associated genera were increased in the IBD/ICS and COPD analyses, respectively.

At the class level, two changes were noted. The Epsilonproteobacteria were increased in the COPD analysis, and this extended to the genus *Campylobacter*. Although usually considered a gastrointestinal pathogen, lung infections have been reported due to aspiration of food [18]. *Campylobacter* was also recently found in the metagenome of cigarettes [19]. The class Synergistia and order Synergistales were increased in the LLQ analysis. These taxa contain the genus *Jonquetella,* a gram-negative anaerobe implicated in periodontal disease and wound infections [20].

At the order level, multiple changes were noted. Coribacteriales, a member of the gram-positive phylum Actinobacteria, were increased in the COPD and LLQ analyses. This increase was extended down to the genus level: *Atopobium,* a vaginal commensal and member of the oral flora [21] and *Cryptobacterium,* a cause of dental abscess [22]. Two orders in the gram-positive phylum Firmicutes were differentially abundant. The Lactobacillales were increased in the COPD and LLQ analyses, and this increase was extended to the genera *Streptococcus* and *Abiotrophia*, well-known members of the oral flora. The Erysipelotrichales were also increased in the COPD analysis. This increase was also reflected at multiple taxonomic levels including the genus *Bulleidia* (*Solobacterium*), an organism implicated in periodontal disease and dental abscesses [23]. The Aeromonadales, an order of the gram-negative class Gammaproteobacteria, were increased in the COPD, ICS, IBD, and Severe (vs. Moderate) COPD analyses. This increase extended to the genus *Aeromonas*, which is found in fresh and brackish water and causes diarrhea and wound infections, particularly in immunocompromised patients [24]. The order Desulfobacterales, in the gram-negative class Deltaproteobacteria, was increased in the COPD, ICS, IBD, and Severe (vs. Moderate) COPD analyses. This increase extended to the genus *Desulfobulbus*, which is associated with periodontitis [25].

Eight additional genera from 4 different phyla were differentially abundant in at least 3 of our 4 analyses. In the phylum Actinobacteria, we noted a decrease in *Humicoccus* and an increase in *Nocardioides*, which have both been found in environmental samples. Within Firmicutes, we noted a decrease in *Thermoactinomyces*, a potential cause of hypersensitivity pneumonitis [26], [27]. Within the phylum Proteobacteria, we saw increases in *Balneimonas* (found in the environment), as well as *Kingella,* a member of the oral flora and a cause of bacteremia and endocarditis [26], [27]. *Citrobacter*, a gastrointestinal pathogen, was decreased. Within the anaerobic phylum Bacteroidetes, we noted increases in the gastrointestinal pathogen *Dysgonomonas* [28] and *Phocaeicola,* a cause of brain abscess [29].

We also performed Metastats analysis comparing our Moderate and Severe COPD samples (Supplementary Table 1). This analysis identified 9 differentially abundant genera, 3 of which were also identified as differentially abundant in our Metastats analysis of control and COPD samples.

## Discussion

Presented here is an analysis of the lung microbiome in 22 patients with Moderate or Severe COPD compared to 10 control patients. This represents the largest analysis of the COPD microbiome yet published, and the only one to primarily include ambulatory patients with moderate or severe disease. Our results indicate a higher level of microbial diversity among the COPD subjects, but this was driven by differences in age between the Moderate and Severe COPD groups. This is in contrast to the findings of Erb-Downward *et al.* [15] in an earlier study of the COPD microbiome, who found that Moderate and Severe COPD patients had little bacterial diversity. We noted that several patients in each of our groups exhibited very low diversity scores, despite obtaining greater than 5,000 sequences per sample and rarefaction curves indicating thorough sampling of the microbiome. It seems likely that a minority of COPD patients and controls exhibit low microbial diversity, and these samples may skew the results of studies with small sample sizes. Our results are consistent with Sze *et al*., who showed that patients with Very Severe COPD maintained greater microbial diversity than control subjects [16]. Our data show that age, rather than severity of COPD, is associated with increased microbial diversity. This analysis excluded the control patients, who were not age-matched to the COPD patients. In our study, Moderate COPD patients were approximately 7 years older than Severe COPD patients. It is unclear to what extent patient age may reflect years since the diagnosis of COPD, as our clinical data do not include the subject’s age at COPD diagnosis.

Our data are consistent with the hypothesis originally proposed by Charlson *et al.* [7] suggesting that the lung microbiome appears to reflect microaspiration of the oral flora. We noted a significant overlap between Control and COPD sample taxa, although our PCoA was able to cluster Control and COPD samples, but not Moderate and Severe COPD samples, separately. Detailed analysis of clinical factors that may account for alterations in the lung microbiome indicated that use of inhaled corticosteroids or inhaled bronchodilators may have accounted for some of the clustering that we observed. The immunomodulatory effects of steroid exposure likely inhibit the immune response to the lung microbiome. This may allow for the persistence or expansion of the lung microbiome. We did not observe clustering based on tobacco exposure, although our study was hampered by relatively few subjects who were actively smoking at the time of bronchoscopy with an average of 17.5 pack-years of tobacco exposure. Based on our data, it does not appear that the microbiome shifted significantly as a result of tobacco exposure. Further longitudinal microbiome studies of smokers both before and after the development of COPD will be needed to address the question of whether or not tobacco exposure alters the microbiome in a way that predisposes smokers to the development of COPD.

A potential weakness of this study is the possibility that nasal or oral contamination of the bronchoscope, and thus the BALF samples, may have contributed to the observed results. This issue was extensively addressed by Charlson *et al.* [7] who noted that the bacteria in the BALF arise from upper respiratory tract bacteria, likely through a combination of microaspiration and bronchoscopic carryover during sampling. Determination of the relative contribution of carryover versus microaspiration on lung microbiome composition will require lung tissue microbiome determination, such as was done by Sze *et al.* [16]. This technique is unfortunately limited to patients who undergo lung explantation or lobectomy, which is not typically performed on patients with relatively stable COPD.

We detected multiple taxa that were differentially abundant in the COPD, inhaled corticosteroid, inhaled bronchodilator and “LLQ” microbiomes. Our findings reinforce the notion that microaspiration of oral flora is the source of the lung microbiome. We identified several common or rare lung pathogens (*Rothia, Tropheryma, Actinomyces, Streptococcus, Peptostreptococcus, Serratia, Capnocytophaga*), as well as known causes of bacteremia or endocarditis (*Rothia, Tropheryma, Streptococcus, Peptostreptococcus, Leptotrichia, Kingella, Dysgonomonas*) among the organisms identified by our analysis. Several anaerobes also were observed, including members of the phyla Fusobacteria, Bacteroidetes, and the genus *Clostridium* within the phylum Firmicutes. Although this is a surprising finding within the presumed aerobic environment of the lung, it is possible that these anaerobes can persist in an abnormal microenvironment of the COPD lung in a manner similar to that seen in lungs affected by cystic fibrosis.

Although other authors have described a “core” lung microbiome, we hesitate to interpret our data in this manner. Both our control and COPD samples included “outlier” samples with very low diversity indices and few OTUs identified despite robust sequencing. Data from healthy controls demonstrated that the lung microbiome composition was much more similar to the same subject’s oral microbiome than to the lung microbiomes of the other subjects [7]. Our data does not support the presence of a “core” microbiome that is stable across multiple subjects, and we suggest that the oral microbiome heavily influences the lung microbiome content.

Both Erb-Downward *et al.* and Sze *et al.* [16] published on the COPD microbiome of patients with Very Severe COPD at the time of lung explantation for lung transplant. Patients presenting for lung transplantation likely experience frequent COPD exacerbations, necessitating frequent systemic steroids and/or broad-spectrum antibiotics. They also likely have very abnormal lung anatomy due to long-standing lung disease. Their abnormal anatomy and use of medications that may alter the microbiome makes it difficult to extrapolate these results to patients with less-severe COPD. Our study is the first to describe the microbiome of a large group of COPD patients whose disease is relatively stable, with no systemic steroid or antibiotic use in the previous 2 months. They represent the best environment in which to study the interactions between the microbiome, the immune system, and COPD pathogenesis, as their disease is still evolving.

Our results, as well as other research on the lung microbiome, indicate that microaspiration of oral flora may serve as the source of the lung microbiome. Multiple bacteria in the COPD microbiome are also found in dental caries, dental abscesses, or periodontal disease. Epidemiologic studies have shown an association between poor oral health and COPD progression. Good oral health and regular professional dental cleaning has improved respiratory outcomes for patients, particularly those living in nursing homes. A recent meta-analysis has shown that periodontal disease may be associated with COPD [30]. As a large proportion of inhaled drugs are retained in the oral cavity, they may also interfere with oral physiology and the oral microbiome. Prolonged use of IBDs is associated with increased dental caries and increased gastroesophageal reflux, while ICSs are associated with increased gingivitis and oral thrush [31]. Ongoing research on the effect of oral health on COPD disease progression and exacerbations will likely further our understanding of the interactions between our oral microbiome, lung microbiome, and the progression of COPD.

## Methods

### Sample Selection

Frozen bronchoalveolar lavage fluid (BALF) samples from 22 patients who participated in the FORTE study were selected for our study [32]. Fourteen of the patients had moderate COPD and 8 had severe COPD, as detailed in Table 1. Patients in this study consented to bronchoscopy at study entry (all samples included in this study were obtained at study entry), and were excluded if they had had smoked or required systemic steroids in the past 6 months or antibiotics in the past 2 months. BALF samples were immediately frozen and maintained at −80°C until thawed for DNA extraction. We also obtained BALF samples from 10 healthy individuals (4 smokers, 6 non-smokers) with normal lung function defined as FEV1>80% predicted and FEV1/FVC >70. Standard clinical protocols were followed to prevent nasopharyngeal contamination of the BALF samples. Per the FORTE study protocol, the nasopharyngeal approach was used preferentially, with the oropharyngeal approach attempted if the nasopharyngeal technique failed. All patients provided informed consent and their identities were not provided to the research team. The institutional review board for human studies approved the protocols (IRB Study 0202M17621 and IRB study 0601E80869).

### DNA Isolation, PCR Amplification, and Sequencing

BALF samples were thawed and 0.5 ml of fluid used for DNA isolation. We used a previously described protocol [33] for DNA isolation that included bead beating to lyse bacterial cells, followed by precipitation with isopropanol and digestion with RNase. Purified DNA was subjected to Multiple Displacement Amplification with REPLI-g (Qiagen, Valencia, CA), which provided highly uniform DNA amplification with minimal amplification bias [34]. REPLI-g was used to minimize PCR cycles, which may introduce bias. PCR amplification using 16S rRNA gene primers specific to the constant regions flanking the V3 region [35], [36] was performed using 20 cycles. The primer sequences were: GCCTCCCTCGCGCCATCAG - 10 base barcode - CCTACGGGAGGCAGCAG 3′ (forward) and 5′ GCCTTGCCAGCCCGCTCAG - ATTACCGCGGCTGCTGG 3′ (reverse). For each sample, a 10 base bar code was included to distinguish patient number and sampling time. Amplicons were gel purified and sequenced at the University of Illinois Urbana-Champaign on a Roche 454 FLX DNA sequencer using titanium chemistry. To minimize effects of random sequencing errors, we used RDP Pipeline [4] to eliminate (a) sequences that did not appropriately match the PCR primer and the barcode at the beginning of a read, (b) sequence reads with <50 bases after the proximal PCR primer if they terminated before reaching the distal primer, and (c) sequences that contained more than one undetermined nucleotide (N). Trim.seqs and chimera.uchime implemented in mothur [37] were used to truncate low-quality sequences and remove chimeras, respectively. Both primers were trimmed from high-quality reads before sequences were submitted to RDP Classifier for taxonomic identification using a bootstrap cutoff of 50%. Operational Taxonomic Units (OTUs) were defined at an identity cutoff of 97% using mothur.

### Data Analysis

For PCoA analyses, sequences were dereplicated and ClustalW was used to align the dereplicated sequences [38]. The aligned sequences were used to generate a phylogenetic tree using Phylip (University of Washington) with a weighted UniFrac distance algorithm [39]. Metastats [40] was used to detect differentially abundant taxa using taxonomic data from RDP Classifier controlling the false discovery rate at 10% for each level of the taxonomy. The Kruskal Wallace test was used to test for differences between the 3 patient groups and *post hoc* comparisons were conducted using the Wilcoxon test with a Bonferroni adjustment for the 3 tests. Multiple linear regression was used to test for differences between patient groups while controlling for the effect of age. All statistical tests were conducted using R version 2.15.

## Supporting Information

Figure S1 **Control and COPD Sample Rarefaction Curves.** Control (top) and COPD patient (bottom) rarefaction curves show that with few exceptions, additional sequencing would not result in discovery of a significant number of additional operational taxonomic units. In the COPD patient rarefaction curve, samples from patients with Severe COPD are indicated with thicker curves.
(TIF)

Figure S2 **Venn Diagram Analysis Demonstrates Significant Overlap Between Moderate and Severe COPD Sample Operational Taxonomic Units (OTU).** A Venn diagram was created using mothur at an OTU similarity cutoff of 97%. All 10 control samples, 14 Moderate COPD samples, and 8 Severe COPD samples were merged into 3 groups–Control (pink), Moderate COPD (green) and Severe COPD (purple). The Control, Moderate COPD, and Severe COPD groups contained 202, 245, and 99 unique OTUs, respectively. Forty-six OTUs were found in all three groups. The largest number (96) of OTUs shared between two groups were shared by the Moderate and Severe COPD groups. In contrast, the Control group shared only 25 and 12 OTUs with the Moderate COPD and Severe COPD groups, respectively.
(TIF)

Figure S3 **Principal Coordinate Analysis Demonstrates No Clustering Based on Percent Emphysema, Age, Gender, or Theophylline Use.** Principal Coordinate Analysis was performed using mothur and Fast UniFrac, and the results for principal coordinates 1 and 2 are shown. A. Percent Emphysema. Percent of lung involved by emphysema was calculated from FORTE study entry chest CT scans. Patients were divided into Low (<25%, yellow), Medium (25–40%, green) and High (>40%, blue) percent emphysema tertiles. Samples do not cluster based on percent emphysema. B. Age. COPD samples were divided by median age. Samples from younger COPD patients (<66 years, yellow) and older COPD patients (≥66 years, blue) did not cluster separately. Control patient samples were not included as our two groups were not age-matched. C. Gender. Control and COPD patients were labeled as male (blue) or female (red). No clustering by gender was observed. D. Theophylline Users and Non-Users. Samples were labeled as theophylline users (blue, 4 of 22 COPD patients) and non-theophylline users (red, 18 of 22 COPD patients and 10 controls). No clustering based on theophylline use was observed.
(TIF)
