# Supplementary figures and images for: The Lung Microbiome in Moderate and Severe Chronic Obstructive Pulmonary Disease
Source: PLoS One. 2012 Oct 11;7(10):e47305. doi: 10.1371/journal.pone.0047305 (PMC3469539; doi:10.1371/journal.pone.0047305)

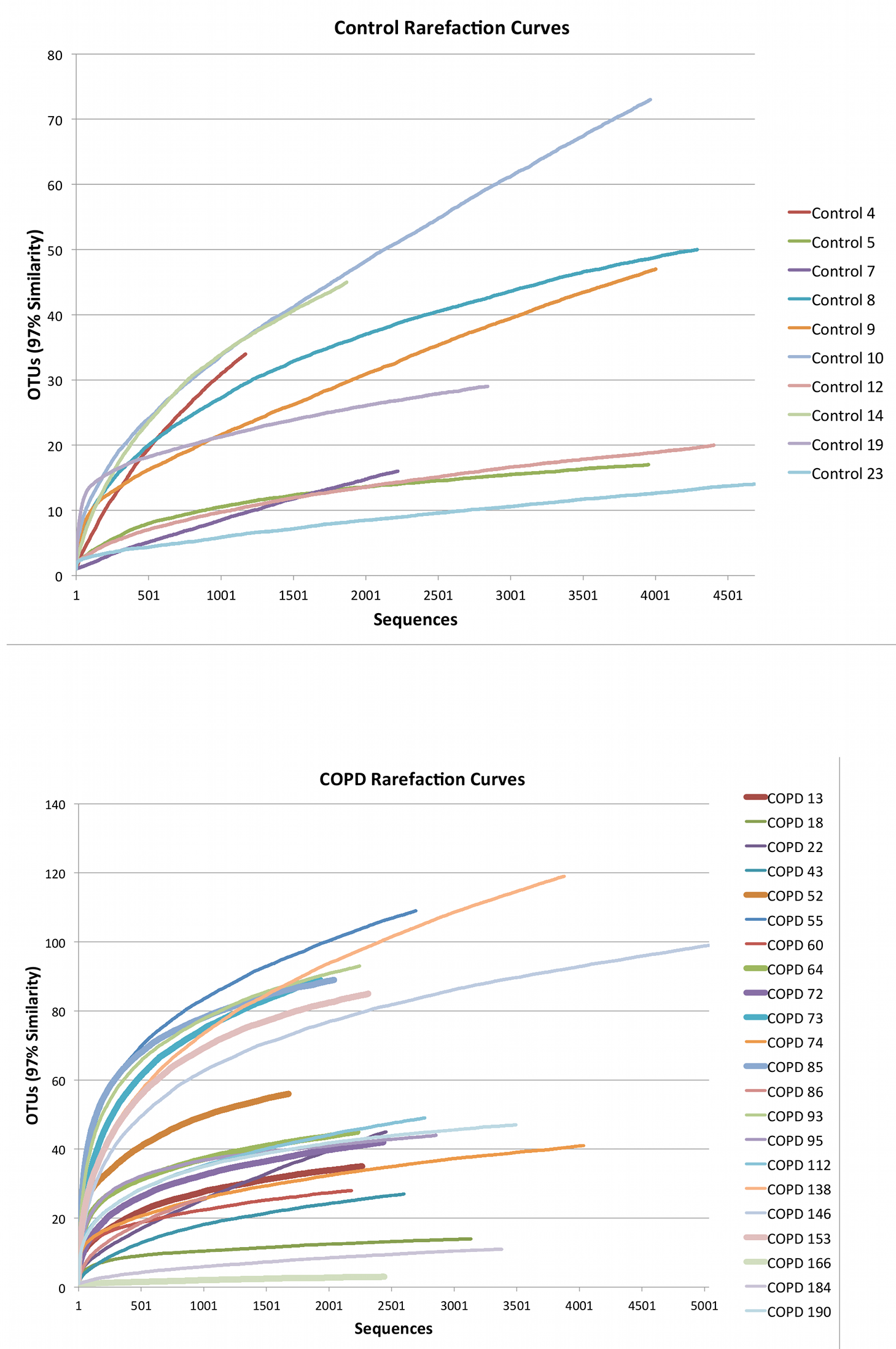

Supplement: Figure S1 — Control and COPD Sample Rarefaction Curves. Control (top) and COPD patient (bottom) rarefaction curves show that with few exceptions, additional sequencing would not result in discovery of a significant number of additional operational taxonomic units. In the COPD patient rarefaction curve, samples from patients with Severe COPD are indicated with thicker curves. (TIF) [file pone.0047305.s001.tif]

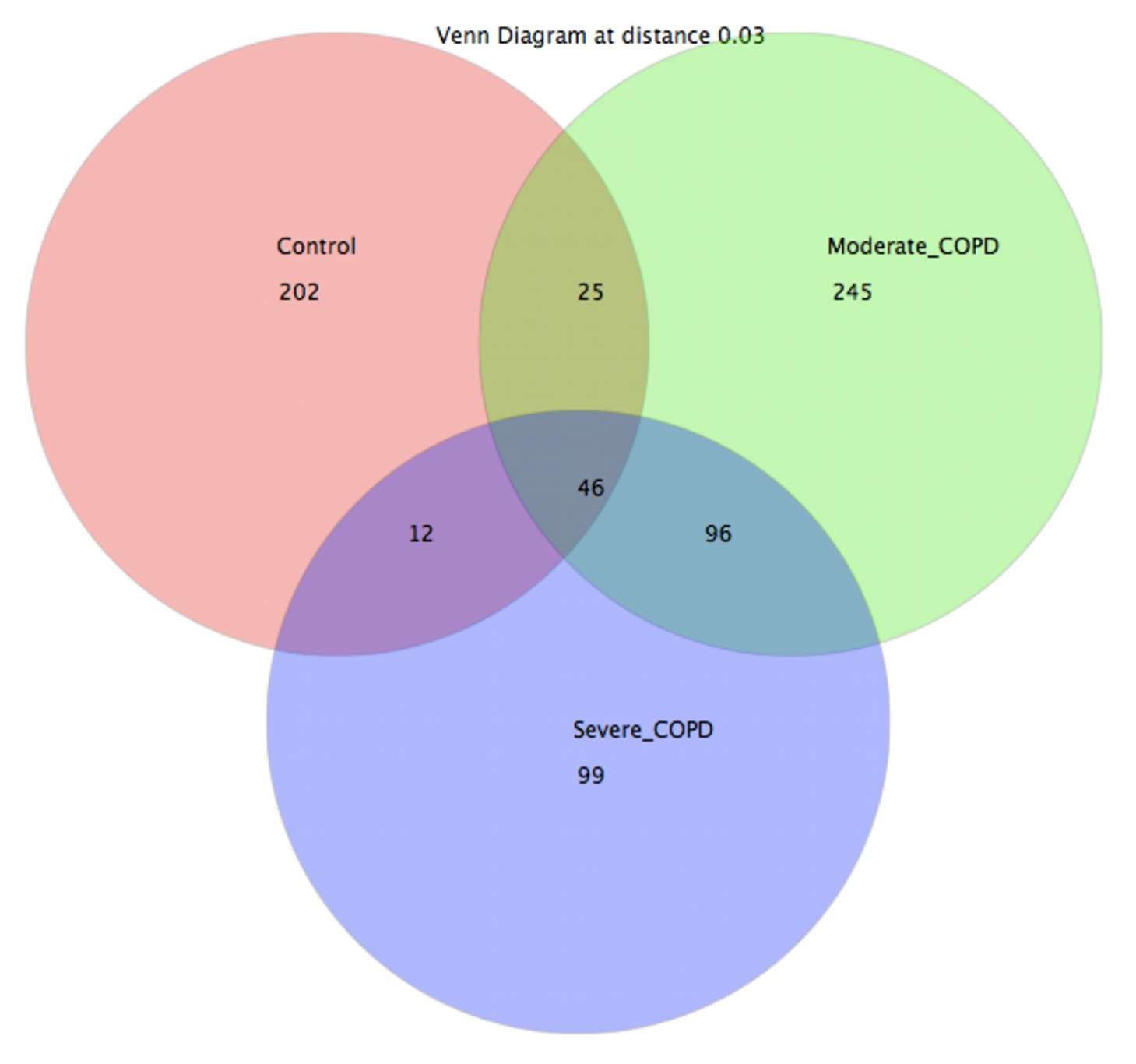

Supplement: Figure S2 — Venn Diagram Analysis Demonstrates Significant Overlap Between Moderate and Severe COPD Sample Operational Taxonomic Units (OTU). A Venn diagram was created using mothur at an OTU similarity cutoff of 97%. All 10 control samples, 14 Moderate COPD samples, and 8 Severe COPD samples were merged into 3 groups–Control (pink), Moderate COPD (green) and Severe COPD (purple). The Control, Moderate COPD, and Severe COPD groups contained 202, 245, and 99 unique OTUs, respectively. Forty-six OTUs were found in all three groups. The largest number (96) of OTUs shared between two groups were shared by the Moderate and Severe COPD groups. In contrast, the Control group shared only 25 and 12 OTUs with the Moderate COPD and Severe COPD groups, respectively. (TIF) [file pone.0047305.s002.tif]

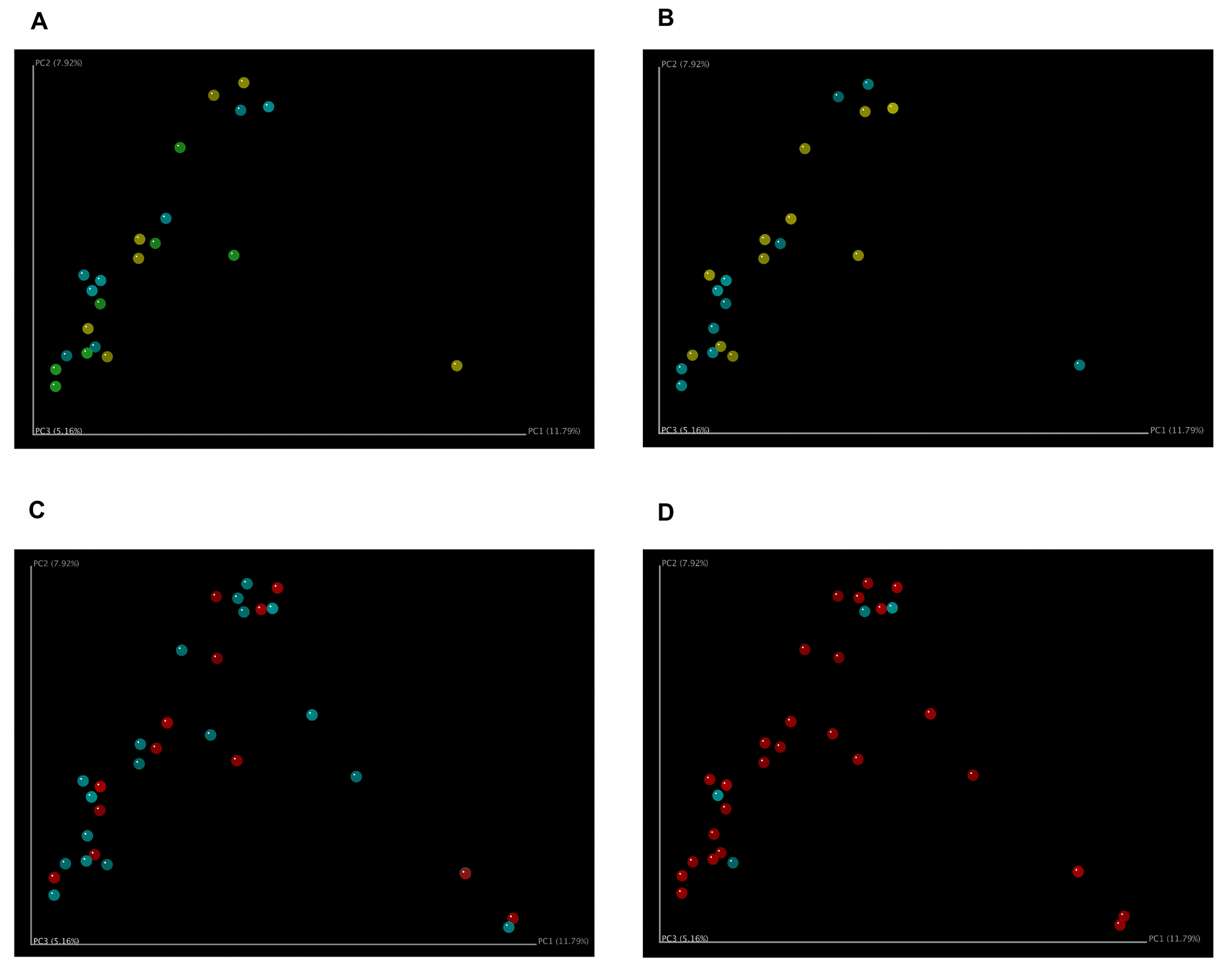

Supplement: Figure S3 — Principal Coordinate Analysis Demonstrates No Clustering Based on Percent Emphysema, Age, Gender, or Theophylline Use. Principal Coordinate Analysis was performed using mothur and Fast UniFrac, and the results for principal coordinates 1 and 2 are shown. A. Percent Emphysema. Percent of lung involved by emphysema was calculated from FORTE study entry chest CT scans. Patients were divided into Low (<25%, yellow), Medium (25–40%, green) and High (>40%, blue) percent emphysema tertiles. Samples do not cluster based on percent emphysema. B. Age. COPD samples were divided by median age. Samples from younger COPD patients (<66 years, yellow) and older COPD patients (≥66 years, blue) did not cluster separately. Control patient samples were not included as our two groups were not age-matched. C. Gender. Control and COPD patients were labeled as male (blue) or female (red). No clustering by gender was observed. D. Theophylline Users and Non-Users. Samples were labeled as theophylline users (blue, 4 of 22 COPD patients) and non-theophylline users (red, 18 of 22 COPD patients and 10 controls). No clustering based on theophylline use was observed. (TIF) [file pone.0047305.s003.tif]
